# Dietary amylose:amylopectin ratio influences the expression of amino acid transporters and enzyme activities for amino acid metabolism in the gastrointestinal tract of goats

**DOI:** 10.1017/S0007114521002087

**Published:** 2022-04-28

**Authors:** Xiaokang Lv, Chuanshe Zhou, Tao Ran, Jinzhen Jiao, Yong Liu, Zhiliang Tan, Shaoxun Tang, Jinhe Kang, Jingjing Xie, Liang Chen, Ao Ren, Qixiang Xv, Zhiwei Kong

**Affiliations:** 1 CAS Key Laboratory of Agro-ecological Processes in Subtropical Region, National Engineering Laboratory for Pollution Control and Waste Utilization in Livestock and Poultry Production, Hunan Provincial Key Laboratory of Animal Nutrition & Physiology and Metabolism, Institute of Subtropical Agriculture, the Chinese Academy of Sciences, Changsha 410125, People’s Republic of China; 2 University of Chinese Academy of Sciences, Beijing 100049, People’s Republic of China; 3 College of Pastoral Agriculture Science and Technology, Lanzhou University, Lanzhou 730020, People’s Republic of China; 4 Department of Animal Science and Technology, University of Hunan Agricultural University, Changsha 410128, People’s Republic of China

**Keywords:** Goats, Amylose, Amylopectin, Protein synthesis, Amino acids transporter, Amino acids metabolism enzyme activity

## Abstract

This study was designed to investigate the effects of dietary starch structure on muscle protein synthesis and gastrointestinal amino acid (AA) transport and metabolism of goats. Twenty-seven *Xiangdong* black female goats (average body weight = 9·00 ± 1·12 kg) were randomly assigned to three treatments, i.e., fed a T1 (normal maize 100 %, high amylose maize 0 %), T2 (normal maize 50 %, high amylose maize 50 %) and T3 (normal maize 0 %, high amylose maize 100 %) diet for 35 d. All AA in the ileal mucosa were decreased linearly as amylose:amylopectin increased in diets (*P* < 0·05). The plasma valine (linear, *P* = 0·03), leucine (linear, *P* = 0·04) and total AA content (linear, *P* = 0·03) increased linearly with the increase in the ratio of amylose in the diet. The relative mRNA levels of solute carrier family 38 member 1 (linear, *P* = 0·01), solute carrier family 3 member 2 (linear, *P* = 0·02) and solute carrier family 38 member 9 (linear, *P* = 0·02) in the ileum increased linearly with the increase in the ratio of amylose in the diet. With the increase in the ratio of amylose:amylopectin in the diet, the mRNA levels of acetyl-CoA dehydrogenase B (linear, *P* = 0·04), branched-chain amino acid transferase 1 (linear, *P* = 0·02) and branched-chain *α*-keto acid dehydrogenase complex B (linear, *P* = 0·01) in the ileum decreased linearly. Our results revealed that the protein abundances of phosphorylated mammalian target of rapamycin (p-mTOR) (*P* < 0·001), phosphorylated 4E-binding protein 1 (*P* < 0·001) and phosphorylated ribosomal protein S6 kinases 1 (*P* < 0·001) of T2 and T3 were significantly higher than that of T1. In general, a diet with a high amylose ratio could reduce the consumption of AA in the intestine, allowing more AA to enter the blood to maintain higher muscle protein synthesis through the mTOR pathway.

## Graphical abstract

**Figure:**
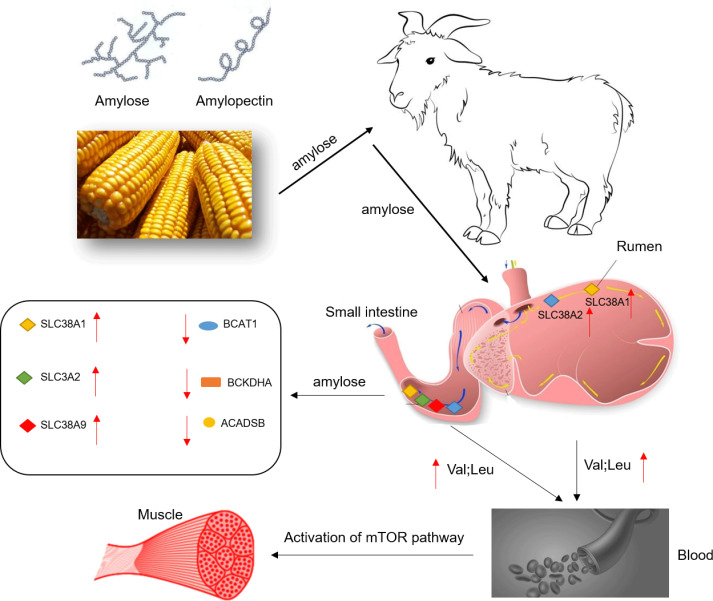

As a widely grown crop, maize provides sufficient carbohydrates for humans and animals and is a primary source of energy^(1)^. Starch is the main component of maize, and it can be separated into two types of glucose polymers due to the difference in glucoside linkage: amylose is a linear chain of glucose units joined by −1,4-glucoside linkages, while amylopectin is a highly branched amylose linked by −1,6 bonds^(2)^. Maize digestibility is mainly determined by starch digestibility, which depends on the amylose:amylopectin ratio and molecular weight^(3)^. Depending on the digestion rate of starch, it can be divided into rapid digestion starch, slow digestion starch and resistant starch^(4)^. The metabolic response and growth performance of pigs also differ when fed diets with different starch digestibilities^(5)^. Due to the slow digestion rate of amylose, the plasma glycaemic level rises slowly after the animal ingests, it could maintain the animal’s postprandial blood glucose homoeostasis^(6,7)^. The level of plasma glycaemic can regulate protein metabolism and amino acid (AA) transport^(8)^. On the other hand, AA are the primary energy source of the small intestines. In addition to participating in protein synthesis, the metabolism of AA in the epithelial cells of the small intestines also provides about 70 % of the energy requirements of the small intestines. Additionally, free AA are usually absorbed through some Na^+^-dependent/Na ^+^-independent AA transport systems located on the apical membrane of intestinal epithelial cells. Simultaneously, AA transport systems can be divided into neutral AA transporters (preferring leucine and other large hydrophobic neutral AA (system L), preferring alanine and other small and polar neutral AA (system A), and preferring alanine, serine and cysteine (system ASC)), anionic AA transporters (X-_AG_ system), cationic AA transporters (y^+^ system) and some individual AA transport vectors (L and T system)^(9)^. However, the relationship between dietary starch structure and AA transport in the gastrointestinal tract of animals has seldom been observed.

Furthermore, previous studies have revealed that the source and type of dietary starch often affect the plasma insulin levels, which in turn affect protein turnover and metabolism in animals^(10–12)^. Due to the faster digestion rate of amylopectin, the plasma insulin also increases rapidly, which is more conducive to the deposition of fat, while the digestion rate of amylose is slower, and the plasma insulin rises slowly and lasts longer, which is more conducive to the lean deposition^(13)^. More protein synthesis is needed in the lean deposition process than the fat. Compared with high amylose diets, animals fed high amylose diets have lower protein digestibility^(14,15)^. It is verified that amylopectin probably reduces the absorption of AA due to its fast digestion^(16,17)^. To our knowledge, information about the influence of dietary starch structure on the absorption and metabolism of AA in the gastrointestinal tract of animals is limited.

Herein, we hypothesised that feeding diets with different amylose:amylopectin ratios would affect the expression of the AA transporters, thus regulate the absorption of AA in the gastrointestinal tract. In this study, goats were thereby used as experimental animals to explore the effects of diets with different amylose:amylopectin ratios on intestinal AA transporters’ expression, the activities of enzymes involved in AA metabolism, and mammalian target of rapamycin (mTOR) signalling pathway involved AA accumulation in muscles, intending to reveal how starch types affect animal protein and AA metabolism.

## Materials and methods

The study received the approval of the Institutional Animal Care Committee, and all procedures involving animals were conducted in accordance with the guidelines on animal care of the Institute of Subtropical Agriculture, the Chinese Academy of Sciences.

### Experimental design, animals and diets

The animal trial was conducted at a commercial Xiangdong black goat farm in Hunan province, P. R. China. Twenty-seven Xiangdong black female goats (average body weight of 9·00 ± 1·12 kg) were selected, blocked by weight and allocated into three groups (*n* 9). Then, the grouped goats were randomly assigned to one of three diets: T1 (normal maize 100 %, high amylose maize 0 %); T2 (normal maize 50 %, high amylose maize 50 %) and T3 (normal maize 0 %, high amylose maize 100 %). The diets were elaborated according to the requirements of the NRC (2007). The ingredients and chemical composition of the concentrate and alfalfa are presented in Table 1. The animal trial lasted 49 d, with 14 d of adaptation period and 35 d of the experimental period. During the experimental period, goats were fed twice daily at 08.00 and 16.00 hours with concentrate and alfalfa offered separately. During the animal trial, all goats had *ad libitum* access to water, concentrate and alfalfa.

**Table 1.** Ingredients and chemical composition of concentrate diets (DM basis, %)

Items	Treatments†		
	T1	T2	T3
Ingredients, %			
Normal maize	83·0	41·5	0
High amylose-maize	0	41·5	83·0
Soyabean meal	12·0	12·0	12·0
CaHPO_4_·2H_2_O	1·5	1·5	1·5
CaCO_3_	0·7	0·7	0·7
Salt	0·8	0·8	0·8
Premix‡	2·0	2·0	2·0
Nutrients, % of DM			
DM (%)	87·6	87·2	87·4
DE (KJ/kg)	15·5	16·0	16·2
CP (%)	11·1	12·0	12·0
Total starch (%)	55·1	50·3	50·3
Amylose/total starch (%)	20·83	37·94	64·70
Amylopectin/total starch (%)	79·17	62·06	35·30
Ca (%)	0·7	0·7	0·8
TP (%)	0·3	0·3	0·3

DE, digestive energy; CP, crude protein; TP, total phosphorus.

Nutritional composition of alfalfa: DM 95·9 %, CP 14·5 %, NDF 37·2 %, ADF 28·1 %.

† T1 (normal maize 100 %, high amylose maize 0 %); T2 (normal maize 50 %, high amylose maize 50 %); T3 (normal maize 0 %, high amylose maize 100 %).

‡ The premix provided the following per kg of the diet: MnSO_4_•H_2_O 15·33 g, FeSO_4_•7H_2_O 30 g, CuSO_4_•5H_2_O 25·33 g, ZnSO_4_•H_2_O 15·33 g, iodine 0·667 g, Se 0·67 g, Co 0·67 g, Vitamin A 32 500 IU, Vitamin D_3_ 10 000 IU, Vitamin E 80 IU, Vitamin K_3_ 10 mg, Vitamin B_1_ 10 mg, Vitamin B_2_ 25 mg, Vitamin B_6_ 8 mg, Vitamin B_12_ 0·075 mg, biotin 0·600 mg, folic acid 5 mg, nicotinamide 100 mg, pantothenic acid 50 mg.

### Sample collection

The blood sample of each goat was collected into a 10-ml vacuum tube (Zhejiang Gongdong Medical Technology Co. Ltd; code No. GD0100LH) containing Na-heparin via the jugular vein before morning feeding on d 30. Plasma samples were prepared via centrifuged at 3000 ***g*** for 20 min at 4°C, subsampled and frozen at −20°C until analysed. The longissimus dorsi and mucosa epithelium samples of the rumen (medium ventral) and ileum (middle) were separated immediately after slaughter and rinsed with cold PBS (0·85 % NaCl, 1·4 mm KH_2_PO_4_, 8 mm Na_2_HPO_4_, pH 7·4). The samples (3 g) were collected from the longissimus dorsi, rumen and ileum, snap-frozen in liquid N_2_ and stored at −80°C until analysis. Meanwhile, tissue samples of the medium ventral of rumen (2 × 2 cm^2^) and the middle part of ileum (5 cm) were collected from the sample location as epithelial samples, washed with PBS, fixed in 10 % formalin (v/v) for 24 h, embedded in paraffin wax and stored at 4°C until use

### Amino acid profile and enzyme activity related to amino acid metabolism

The free AA profiles of intestinal epithelial, plasma and longissimus dorsi samples were determined according to the method described by Li *et al.* ^(18)^. The activities of enzymes involved in AA metabolism, including branched-chain amino acid transferase (BCAT), branched-chain *α*-keto acid dehydrogenase complex (BCKDH), acetyl-CoA dehydrogenase (ACADS) and short/branched-chain ACAD (ACADSB), were determined using commercial ELISA kits (goat#ml567708, goat#ml601711, goat#ml211443, goat#ml221113, Mlbio, Shanghai, China, and CW0014, CWBIO, Beijing, China) following the recommended procedures.

### Amino acid transporter and receptor expression

The relative mRNA levels of AA transporters were determined using quantitative real-time PCR. Briefly, total RNA was extracted from collected epithelial samples using RNAiso Plus (TaKaRa; Code No. 9108/9109) following the manufacturer’s instructions. The genomic DNA was eliminated by digestion with DNase I (Thermo Scientific), and then the purity and concentration of total RNA were measured by NanoDrop 2000 (Thermo Scientific). Afterwards, 1 μg total RNA was reverse-transcribed to cDNA in a 20 μl system using the Evo M-MLV RT Kit (AG11706) following the manufacturer’s instructions. The synthesised cDNA was saved at −20°C until used for quantitative real-time PCR analysis. Real-time quantitative PCR was performed using the SYBR Premix Ex Taq II (Takara) on an ABI-7900HT qPCR system (Applied Biosystems), with *β*-actin selected as housekeeping genes^(19)^. All primers were synthesised commercially (Sangon Biotech), and the primer sequences for the target genes are shown in Supplementary Table S1. The relative expression levels of mRNA were estimated according to the 2^−ΔΔCT^ method^(20)^.

### Immunofluorescent analysis of amino acid transporters

Embedded rumen and ileum tissue samples were cut into 4 µm sections with a cryostat microtome (Leica CM1850, Leica Microsystems). Slides were dewaxed and blocked with 2 % BSA at room temperature for 1 h. Subsequently, the slides were incubated with primary antibodies at 4°C overnight. Following incubation, sections were washed five times for 3 min each time in PBS and incubated with the secondary antibody for 1 h at room temperature. Subsequently, slides were washed for 5 × 3 min in PBS and stained with 4',6-diamidino-2-phenylindole solution for 8 min. The slides were furtherly stained with the configured Sudan black dye solution (70 ml 70 % ethanol + 30 ml pure water + 0·3 g Sudan black) for 5 min and rinsed under running distilled water for 20 min. Immunostained slides were examined with a Zeiss LSM880 confocal microscope (Carl-Zeiss-Straße22, 73447Oberkochen) with argon and He-Ne laser sources. On average, eight images were taken per section. Image processing and analysis were conducted on ImageProline Plus 5.1 (Media Cybernetics), and exposure time and gain between all images on each slide were kept constant. Primary and corresponding secondary antibodies and working dilutions are shown in Supplementary Table S2.

### Western blot analysis

Western blot analysis was carried out to analyse the relative protein abundances of mTOR, 4EBP1 (4E-binding protein 1), S6K1 (ribosomal protein S6 kinases 1) and their phosphorylated forms p-mTOR, p-4EBPI and p-S6K1 in the longissimus dorsi muscle of goats according to previous studies^(21)^. Briefly, total proteins were extracted from the longissimus dorsi muscle samples using RIPA lysate (Applygen Technologies), with 1 % protease inhibitor cocktail (Roche Diagnostics GmbH), followed by 30 min of cleavage on ice. Supernatants were taken after centrifuging at 12 000 ***g*** for 15 min at 4°C, and then the protein concentrations were measured utilising a BCA Protein Assay kit (Hin Biotech). The exact amounts of protein needed for each sample were calculated and mixed with 5× loading buffer, incubated at 95°C for 5 min and stored at –20°C until further analysis. Fifty milligrams of each sample’s total protein was loaded and separated by electrophoresis in 10 % SDS-PAGE gel. The protein spots were then transferred to the PVDF film, which was then incubated with 5 % skimmed milk in a TBS buffer containing 0·2 % Tween-20 for 1 h to suppress the nonspecific binding of Ig. Then, the pre-blocked membrane was incubated with primary antibodies in 1 × TBST at 4°C overnight, washed in 1 × TBST three times (15 min each), incubated with horseradish peroxidase-labelled secondary antibody in 1 × TBST for 1 h at room temperature and washed in 1 × TBST (3 × 10 min). The antibody of GAPDH was used as an internal control to normalise the data. The resultant signals were quantified by Image Processing Software (Image-Proline Plus 6.0). Details of the antibodies used are shown in Supplementary Table S3.

### Statistical analysis

All statistical analyses and visualisation were conducted using SAS version 9.4 (SAS Institute, Inc.) and GraphPad Prism 8.0 (GraphPad Software), respectively. All data analyses were performed by one-way ANOVA with the SAS statistical software (SAS Inc.). Polynomial contrasts were used to determine the linear and quadratic effects of dietary amylose levels. A *P*-value < 0·05 was considered to indicate a statistically significant difference, and a tendency was considered at 0·05 *≤ P* < 0·10.

## Results

### Gastrointestinal mucosa, plasma and longissimus dorsi amino acid profiles

The majority of the free AA (except glycine and alanine) and total AA in ruminal mucosa quadratically (*P* < 0·05) or tended (0·5 < *P* < 0·10) to be quadratically changed with the increment of dietary amylose:amylopectin ratios (online Supplementary Table S4), with AA and total AA concentration increased from T1 to T2 and decreased from T2 to T3. All AA in the ileal mucosa decreased linearly (*P* < 0·05) as amylose:amylopectin increased in diets (Table 2), with greater concentration in T1 than in T3. Furthermore, the concentration of plasma alanine (*P* = 0·001), valine (*P* = 0·03), leucine (*P* = 0·04) and total AA (*P* = 0·03) linearly increased when the amylose:amylopectin ratio increased, with greater concentration observed in T3 than that in T1 (Table 3), whereas the remaining AA did not differ among diets. The concentration of aspartate (*P* = 0·005), alanine (*P* = 0·03), leucine (*P* = 0·009) and proline (*P* = 0·005) in the longissimus dorsi increased linearly when the amylose:amylopectin ratio increased.

**Table 2.** Effects of different amylose:amylopectin ratios on amino acid profiles in ileum mucosal of goats (Mean values and standard errors of the mean)

Items*	Treatments†			sem	*P*-value‡	
	T1	T2	T3		*L*	*Q*
Aspartate	1·09	1·00	0·93	0·02	0·004	0·83
Threonine	0·58	0·53	0·49	0·01	0·002	0·90
Serine	0·57	0·51	0·49	0·01	0·003	0·38
Glutamate	1·73	1·59	1·47	0·04	0·002	0·87
Glycine	0·96	0·92	0·78	0·02	< 0·001	0·17
Alanine	0·87	0·82	0·73	0·02	0·001	0·46
Valine	0·72	0·67	0·61	0·02	0·002	0·92
Methionine	0·25	0·23	0·20	0·01	0·003	0·65
Isoleucine	0·55	0·51	0·48	0·01	0·010	0·77
Leucine	1·10	1·01	0·94	0·03	0·008	0·86
Tyrosine	0·44	0·42	0·38	0·01	0·03	0·50
Phenylalanine	0·59	0·54	0·50	0·01	0·009	0·90
Lysine	1·15	1·03	0·97	0·03	0·007	0·60
Histidine	0·31	0·28	0·26	0·01	0·004	0·64
Arginine	1·09	1·01	0·93	0·02	0·005	0·93
Proline	0·75	0·71	0·62	0·02	0·001	0·43
TAA	12·74	11·77	10·78	0·28	0·003	0·99

TAA, total amino acids.

* (DM basis, %).

† Treatments were: T1 (normal maize 100 %, high amylose maize 0 %); T2 (normal maize 50 %, high amylose maize 50 %); T3 (normal maize 0 %, high amylose maize 100 %).

‡ *L* and *Q* represent linear and quadratic response to increasing amylose:amylopectin ratio.

**Table 3.** Effects of different amylose:amylopectin ratios on plasma amino acid profiles in goats (Mean values and standard errors of the mean)

Items*	Treatments†			sem	*P*-value‡	
	T1	T2	T3		*L*	*Q*
Aspartate	5·23	5·83	5·43	0·29	0·79	0·43
Threonine	26·22	22·00	27·55	0·96	0·54	0·01
Serine	10·42	9·03	11·10	0·44	0·52	0·07
Glutamate	36·72	40·72	39·68	1·20	0·33	0·33
Glycine	65·16	70·05	75·32	2·32	0·06	0·71
Alanine	22·53	27·78	30·61	1·07	0·001	0·92
Valine	20·28	27·99	31·36	1·48	0·03	0·70
Methionine	3·75	4·28	4·57	0·23	0·16	0·81
Isoleucine	11·82	12·40	14·92	0·67	0·06	0·48
Leucine	15·60	19·68	22·22	1·19	0·04	0·75
Tyrosine	17·14	16·28	20·43	1·12	0·24	0·30
Phenylalanine	10·69	10·26	12·11	0·46	0·21	0·24
Lysine	20·24	24·72	24·52	1·03	0·09	0·27
Histidine	8·2	9·38	9·53	0·35	0·12	0·48
Arginine	38·89	38·97	43·17	1·30	0·19	0·46
Proline	12·73	13·28	14·53	0·47	0·13	0·72
TAA	320·19	351·65	386·19	10·25	0·03	0·73

TAA, total amino acids.

* (DM basis, %).

† Treatments were: T1 (normal maize 100 %, high amylose maize 0 %); T2 (normal maize 50 %, high amylose maize 50 %); T3 (normal maize 0 %, high amylose maize 100 %).

‡ *L* and *Q* represent a linear and quadratic response to increasing amylose to amylopectin ratio.

**Table 4.** Effects of different amylose:amylopectin ratios on longissimus dorsi amino acid profiles in goats (Mean values and standard errors of the mean)

Items*	Treatments†			sem	*P*-value‡	
	T1	T2	T3		*L*	*Q*
Aspartate	6·05	6·67	6·65	0·10	0·005	0·07
Threonine	3·32	3·59	3·58	0·06	0·08	0·26
Serine	2·33	2·51	2·51	0·04	0·08	0·32
Glutamate	11·36	11·06	11·27	0·14	0·79	0·41
Glycine	4·14	4·17	4·04	0·06	0·55	0·57
Alanine	4·05	4·45	4·56	0·10	0·03	0·46
Valine	3·38	3·55	3·66	0·07	0·27	0·18
Methionine	1·56	1·64	1·63	0·05	0·54	0·71
Isoleucine	3·45	3·64	3·60	0·06	0·33	0·42
Leucine	5·94	6·48	6·66	0·12	0·009	0·43
Tyrosine	2·17	2·31	2·32	0·05	0·21	0·55
Phenylalanine	3·16	3·33	3·25	0·05	0·48	0·27
Lysine	6·07	6·04	6·08	0·06	0·95	0·76
Histidine	2·13	2·29	2·14	0·06	0·94	0·26
Arginine	5·42	5·33	5·14	0·06	0·06	0·69
Proline	2·69	3·53	3·34	0·12	0·005	0·01
TAA	67·23	70·68	70·31	0·77	0·10	0·24

TAA, total amino acids.

* (DM basis, %).

† Treatments were: T1 (normal maize 100 %, high amylose maize 0 %); T2 (normal maize 50 %, high amylose maize 50 %); T3 (normal maize 0 %, high amylose maize 100 %).

‡ *L* and *Q* represent a linear and quadratic response to increasing amylose to amylopectin ratio.

### Relative mRNA levels of amino acid transporters and receptors

The relative mRNA levels of various AA transporters and receptors in the ruminal and ileal mucosal of goats with a response to different dietary amylose:amylopectin ratios are shown in Table 5. The mRNA levels of Ca-sensing receptor, metabotropic glutamate receptor 7, solute carrier family 7 member 1, solute carrier family 7 member 5 (except a quadratic response in the ileum) and solute carrier family 7 member 10 were not affected (*P* > 0·05) by dietary amylose:amylopectin ratios, whereas the mRNA levels of the remaining AA transporters were affected by dietary amylose:amylopectin ratios in at least one gastrointestinal section, among which the expression of solute carrier family 38 member 1 (SLC38A1), solute carrier family 38 member 2 (SLC38A2) and solute carrier family 38 member 9 (SLC38A9) was most sensitive to the dietary amylose:amylopectin ratio. Quadratic (*P* < 0·05) responses of the mRNA level of solute carrier family 1 member 3 in the rumen and solute carrier family 1 member 4 in the rumen and ileum were observed; the mRNA level of solute carrier family 3 member 2 (SLC3A2) in the ileum increased linearly with dietary amylose:amylopectin ratio, with greater expression in T2 and T3 than in T1. The mRNA level of SLC38A1 in the rumen quadratically changed (*P* = 0·02) with increasing dietary amylose:amylopectin ratio, with greater expression in T2 than in T1, while its level in the ileum increased linearly (*P* = 0·01) with dietary amylose:amylopectin ratio increased, with greater expression in T2 and T3 than in T1. The mRNA level of SLC38A2 in the rumen increased linearly (*P* = 0·03) with increasing dietary amylose:amylopectin ratio, with greater expression noted in T3 than in T1. Similarly, the mRNA level of SLC38A9 in the ileum increased linearly (*P* = 0·02) as the dietary amylose:amylopectin ratio increased, with greater expression in T2 and T3 than in T1.

**Table 5.** Effects of different amylose:amylopectin ratios on mRNA expression of amino acid transporters in ruminal and ileal mucosal of goat (Mean values and standard errors of the mean)

Items	Section	Treatments*			sem	*P*-value†	
		T1	T2	T3		*L*	*Q*
CASR	Rumen	0·90	0·73	0·80	0·09	0·30	0·40
	Ileum	0·87	0·73	0·61	0·06	0·33	0·84
GRM7	Rumen	1·19	1·06	0·99	0·12	0·51	0·92
	Ileum	0·98	0·87	0·74	0·10	0·28	0·39
SLC1A1	Rumen	0·98	1·09	1·14	0·04	0·16	0·82
	Ileum	1·18	0·74	0·83	0·11	0·12	0·57
SLC1A3	Rumen	1·04	1·74	1·24	0·17	0·65	0·10
	Ileum	1·05	0·68	0·83	0·06	0·07	0·02
SLC1A4	Rumen	1·12	2·50	1·79	0·21	0·17	0·01
	Ileum	1·01	0·83	1·26	0·05	0·01	0·01
SLC3A2	Rumen	1·05	0·89	1·05	0·10	0·99	0·49
	Ileum	1·05	1·98	1·85	0·13	0·02	0·12
SLC7A1	Rumen	1·29	0·94	1·31	0·26	0·46	0·93
	Ileum	1·22	0·99	0·99	0·14	0·52	0·71
SLC7A5	Rumen	0·89	1·07	1·12	0·07	0·21	0·66
	Ileum	1·11	0·69	1·13	0·10	0·95	0·03
SLC7A10	Rumen	0·88	0·90	0·98	0·10	0·73	0·91
	Ileum	0·95	0·76	0·82	0·07	0·42	0·42
SLC38A1	Rumen	1·03	2·39	1·18	0·27	0·80	0·02
	Ileum	1·02	1·17	1·55	0·08	0·01	0·48
SLC38A2	Rumen	0·70	0·95	1·22	0·09	0·03	0·94
	Ileum	0·93	1·18	0·82	0·07	0·54	0·05
SLC38A9	Rumen	1·15	1·14	1·08	0·10	0·77	0·93
	Ileum	0·92	1·63	1·72	0·13	0·02	0·30

GRM7, glutamate receptor, metabotropic 7; CASR, Ca-sensing receptor; SLC1A1, solute carrier family 1 member 1; SLC1A3, solute carrier family 1 member 3; SLC1A4, solute carrier family 1 member 4; SLC7A5, solute carrier family 7 member 5; SLC7A10, solute carrier family 7 member 10; SLC38A1, solute carrier family 38 member 1; SLC38A2, solute carrier family 38 member 2; SLC7A1, solute carrier family 7 member 1; SLC3A2, solute carrier family 3 member 2; SLC38A9, solute carrier family 38 member 9.

* Treatments were: T1 (normal maize 100 %, high amylose maize 0 %); T2 (normal maize 50 %, high amylose maize 50 %); T3 (normal maize 0 %, high amylose maize 100 %).

† *L* and *Q* represent linear and quadratic response to increasing amylose:amylopectin ratio.

### Immunofluorescent analysis of solute carrier family 38 member 1, solute carrier family 38 member 2, solute carrier family 38 member 9 and solute carrier family 3 member 2

**Fig. 1.**
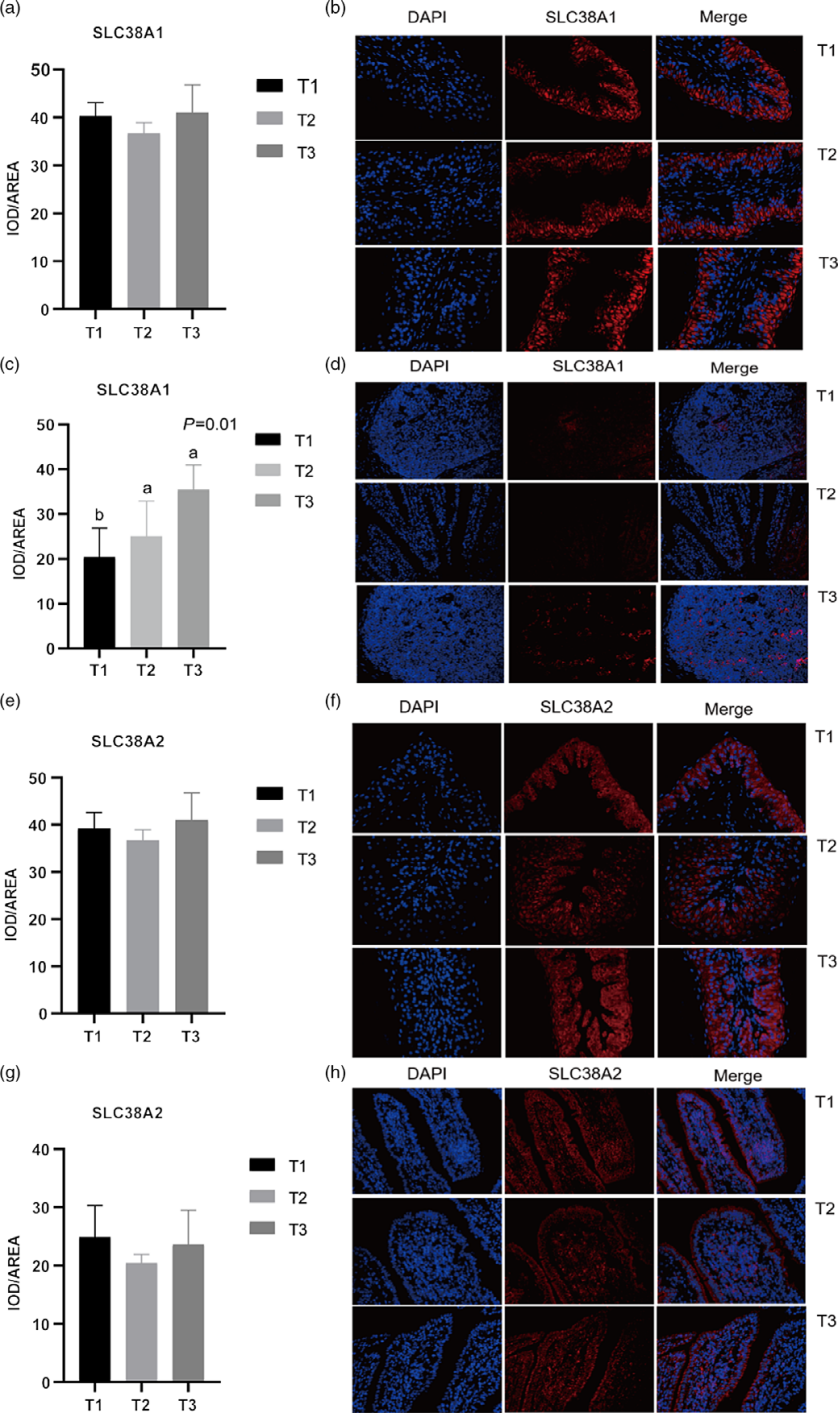
Mean optical density and representative staining of solute carrier family 38 member 1 (SLC38A1) in the rumen (a), (b) and ileum (c), (d); mean optical density and representative staining of solute carrier family 38 member 2 (SLC38A2) in the rumen (e), (f) and ileum (g), (h). All photos were taken at 400×, with nuclei stained in blue and target protein stained in red. Treatments were: T1 (normal maize 100%, high amylose maize 0%); T2 (normal maize 50 %, high amylose maize 50 %); T3 (normal maize 0 %, high amylose maize 100 %); Statistical significance was accepted at *P* < 0·05.

**Fig. 2.**
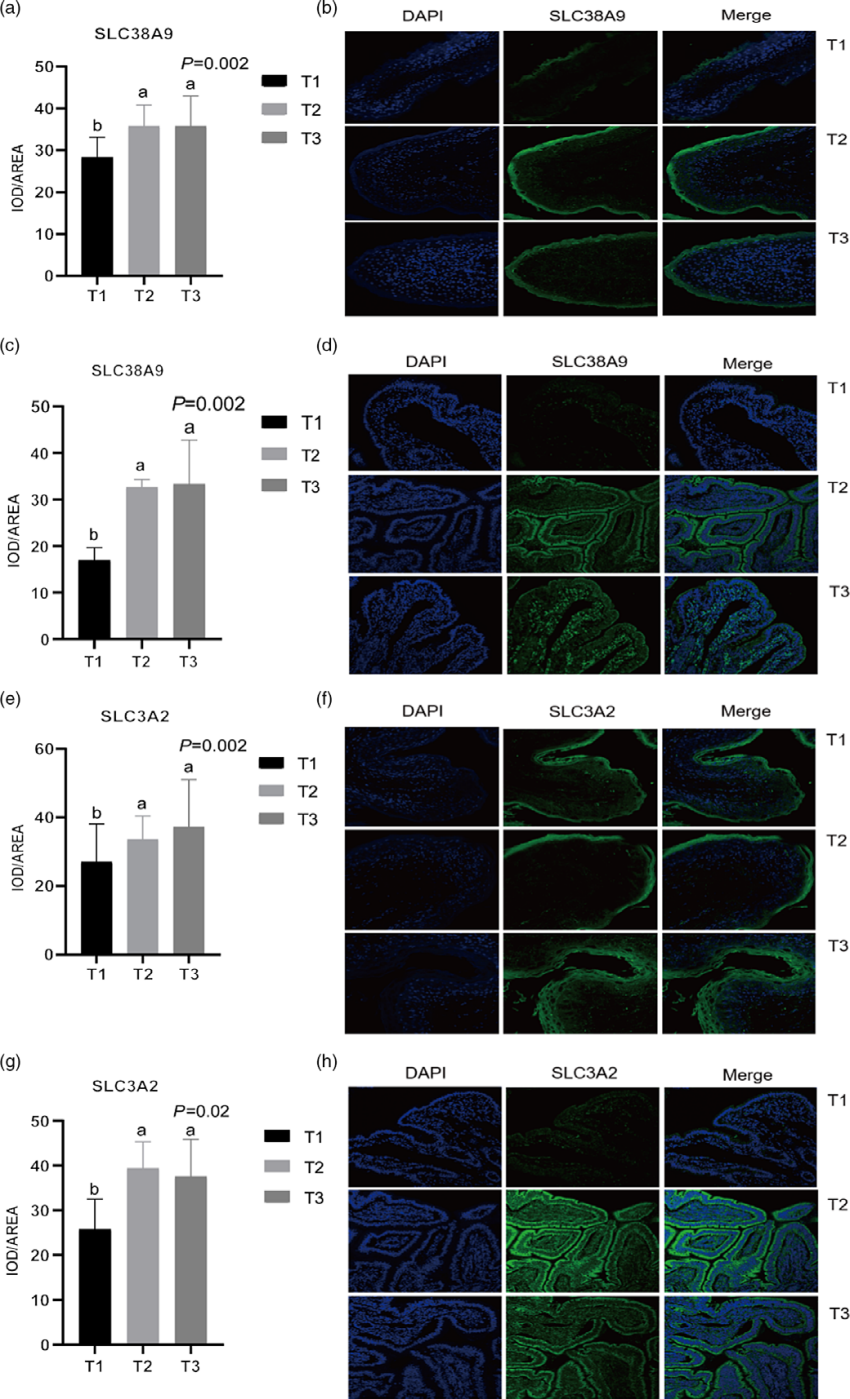
Mean optical density and representative staining of solute carrier family 38 member 9 (SLC38A9) in the rumen (a), (b) and ileum (c), (d); mean optical density and representative staining of solute carrier family 3 member 2 (SLC3A2) in the rumen (e), (f) and ileum (g), (h). T1 (normal maize 100 %, high amylose maize 0 %); T2 (normal maize 50 %, high amylose maize 50 %); T3 (normal maize 0 %, high amylose maize 100 %). Statistical significance was accepted at *P* < 0·05.

**Fig. 3.**
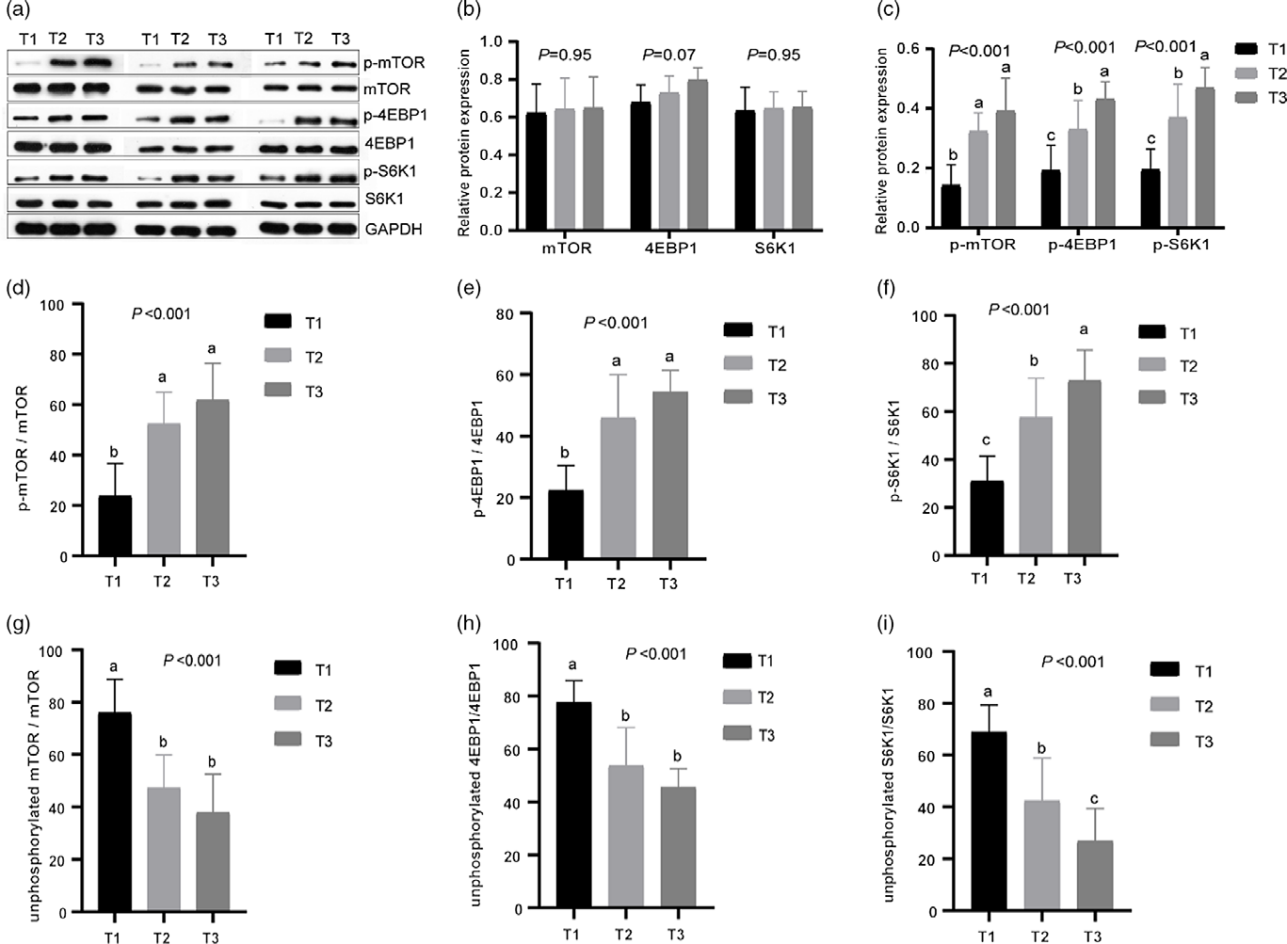
Effects of different dietary amylose:amylopectin ratios on protein expression of phosphorylated and unphosphorylated mammalian target of rapamycin (mTOR), 4E-binding protein 1 (4EBPI), ribosomal protein S6 kinases 1 (S6K1) in the longissimus muscle of goat. (a) Representative lanes of Western blot analysis; (b) relative protein expression of mTOR, 4EBP1 and S6K1; (c) relative protein expression of phosphorylated mammalian target of rapamycin (p-mTOR), phosphorylated 4E-binding protein 1 (p-4EBP1) and phosphorylated ribosomal protein S6 kinases 1 (p-S6K1); (d), (f) phosphorylation ratio of mTOR, 4EBP1 and S6K1. (g), (i) Unphosphorylation ratio of mTOR, 4EBP1 and S6K1. T1 (normal maize 100%, high amylose maize 0 %); T2 (normal maize 50 %, high amylose maize 50 %); T3 (normal maize 0 %, high amylose maize 100 %). ^a,b,c^Mean column with different superscripts differ (*P* < 0·05).

Given the relative mRNA levels of SLC38A1, SLC38A2 and SLC38A9 were significantly different among diets in the rumen and ileum, their protein abundance and localisation were furtherly analysed using immunofluorescence (Fig. 1 and Fig. 2). Immunolocalisation showed that SC38A1 and SLC38A2 were abundantly expressed in the lamina propria, basal layer, and papillae of the rumen, whereas less abundantly expressed in the ileum. The protein abundance of SLC38A1 in the ileum of T3 and T2 was higher (*P* < 0·05) than that of T1 (Fig. 2(c) and (d)), while the protein abundance of SLC38A2 in the ileum was not affected by dietary amylose:amylopectin ratio among treatments. The SLC38A9 and SLC3A2 were both expressed in the rumen mucosa, and their protein abundance was elevated remarkably (*P* < 0·05) in T2 and T3 compared with that of T1 (Fig. 3(a), (b), (e), (f)). Similarly, the protein abundance of SLC38A9 (*P* < 0·05) and SLC3A2 (*P* < 0·05) was enhanced significantly in the ileal mucosa in T2 and T3 compared with T1 due to increased dietary amylose:amylopectin ratios (Fig. 3(c), (d), (g), (h)).

### Relative mRNA levels of branched-chain amino acid metabolism enzymes and their activities

The mRNA levels of the branched-chain AA (BCAA) metabolising enzymes, including ACADSB, BCAT1, BCAT2, BCKDHA, BCKDHB and ACADS, in the ruminal mucosa were not altered (*P* > 0·05) by dietary amylose:amylopectin ratios (Table 6). However, the mRNA levels of ACADSB (linear, *P* = 0·04), BCAT1 (quadratic, *P* = 0·02) and BCKDHB (linear, *P* = 0·01) in the ileum decreased significantly with the increment of dietary amylose:amylopectin ratios, with greater (*P* < 0·05) expression in T1 than in T2 and T3

**Table 6.** Effects of different amylose:amylopectin ratios on relative mRNA expression of branched-chain amino acid metabolising enzymes in the ruminal and ileal mucosal of goat (Mean values and standard errors of the mean)

Section	Items*	Treatments†			sem	*P*-value‡	
		T1	T2	T3		*L*	*Q*
Rumen	BCAT1	1·36	1·41	1·24	0·23	0·85	0·84
	BCAT2	1·12	1·16	1·08	0·21	0·77	0·65
	BCKDHA	1·04	1·13	1·06	0·19	0·54	0·31
	BCKDHB	1·07	1·04	1·60	0·12	0·11	0·23
	ACADS	1·24	1·03	0·87	0·18	0·45	0·95
	ACADSB	1·17	0·90	1·07	0·15	0·82	0·53
Ileal	BCAT1	1·23	0·26	0·59	0·13	0·02	0·005
	BCAT2	1·04	1·11	1·09	0·10	0·33	0·29
	BCKDHA	1·16	1·12	1·05	0·09	0·44	0·38
	BCKDHB	1·13	0·74	0·70	0·07	0·01	0·23
	ACADS	1·05	1·12	0·90	0·08	0·45	0·43
	ACADSB	1·06	0·58	0·68	0·08	0·04	0·08

* BCKDHA, branched-chain *α*-keto acid dehydrogenase E1, *α* polypeptide; BCKDHB, branched-chain keto acid dehydrogenase E1 subunit beta; ACADS, acyl-CoA dehydrogenase short-chain; ACADSB, acyl-CoA dehydrogenase short/branched-chain; BCAT1, branched-chain AA transaminase 1; BCAT2, branched-chain amino acid transaminase 2.

† Treatments were: T1 (normal maize 100 %, high amylose maize 0 %); T2 (normal maize 50 %, high amylose maize 50 %); T3 (normal maize 0 %, high amylose maize 100 %).

‡ *L* and *Q* represent linear and quadratic response to increasing amylose:amylopectin ratio.

**Table 7.** Effects of different amylose:amylopectin ratios on activities of branched-chain amino acid metabolising enzymes (U/mg of protein) in the rumen and ileum of goat (Mean values and standard errors of the mean)

Section	Items*	Treatments†			sem *Q*	*P*-value‡	
		T1	T2	T3		*L*	*Q*
Rumen	ACADSB	5·48	5·44	5·57	0·23	0·89	0·88
	BCAT	2·18	2·03	1·85	0·10	0·17	0·95
	BCKDH	9·61	9·22	10·02	0·35	0·64	0·48
	ACADS	13·25	12·24	12·86	0·36	0·66	0·34
Ileal	ACADSB	5·35	4·52	4·30	0·18	0·003	0·34
	BCAT	2·32	1·63	1·75	0·10	0·002	0·007
	BCKDH	7·55	7·52	6·97	0·19	0·23	0·56
	ACADS	10·11	10·66	9·89	0·29	0·76	0·32

* BCKDH, branched-chain keto acid dehydrogenase complex; ACADS, acyl-CoA dehydrogenase short-chain; ACADSB, acyl-CoA dehydrogenase short/branched-chain; BCAT1, branched-chain AA transaminase 1; One unit of enzyme activity was defined as ……. mg of protein.

† Treatments were: T1 (normal maize 100 %, high amylose maize 0 %); T2 (normal maize 50 %, high amylose maize 50 %); T3 (normal maize 0 %, high amylose maize 100 %).

‡ *L* and *Q* represent the linear and quadratic response to increasing amylose:amylopectin ratio.

The activity of BCAA metabolising enzymes in the rumen and ileum was measured, and there were no differences (*P*>0·05) in enzyme activity in the rumen mucosa among treatments (Table 7). The activities of ACADSB and BCAT in the ileal mucosa decreased linearly (*P* < 0·05) as dietary amylose:amylopectin increased.

**Table 8.** Effects of different dietary amylose:amylopectin ratios on the expression of genes related to muscle protein synthesis of goat (Mean values and standard errors of the mean)

Items*	Treatments†			sem	*P*-value‡	
	T1	T2	T3		*L*	*Q*
mTOR	0·98	1·54	1·50	0·11	0·04	0·17
4EBP1	1·14	1·79	2·08	0·11	0·001	0·32
S6K1	1·02	1·72	1·74	0·12	0·01	0·15

* mTOR, mammalian target of rapamycin; 4EBPI, 4E-binding protein 1; S6K1, ribosomal protein S6 kinases 1.

† Treatments were: T1 (normal maize 100 %, high amylose maize 0 %); T2 (normal maize 50 %, high amylose maize 50 %); T3 (normal maize 0 %, high amylose maize 100 %).

‡ *L* and *Q* represent linear and quadratic response to increasing amylose:amylopectin ratio.

### The mRNA levels and protein abundances of mammalian target of rapamycin, 4E-binding protein 1 and ribosomal protein S6 kinases 1

Expression of mTOR, 4EBP1 and S6K1 in longissimus dorsi muscle was measured at mRNA and protein level (Table 8 and Fig. 3). The mRNA levels of mTOR, 4EBP1 and S6K1 increased linearly (*P* < 0·05) with increasing dietary amylose contents, with greater values observed in T2 and T3 than in T1 (Table 2). The protein abundances of mTOR, 4EBP1 and S6K1 did not differ (*P* > 0·05) among treatments; however, the relative protein abundances of p-mTOR, p-4EBP1 and p-S6K1 increased linearly (*P* < 0·01) with increasing dietary amylose contents, with greater p-mTOR in T2 and T3 than in T1, and greater p-4EBP1 and S6K1 in T3 than in T2 and T1 (Fig. 3). Therefore, the phosphorylated:non-phosphorylated ratio of mTOR, 4EBP1 and S6K1 was elevated significantly (*P* < 0·01) in T2 and T3 compared with T1.

## Discussion

The rumen and small intestines are the main places for digestion and absorption of nutrients in ruminants. Previous studies have revealed that the type of starch in the diets can affect the body’s energy and protein metabolism^(10,12,22)^. In the current study, we found that increasing the ratio of amylose in the diet could reduce the deposition of AA in the ileal mucosa of goats, but increase the content of leucine, valine, tryptophan and total AA in the plasma, which suggests that more AA would be transported from the mucosa to the blood to maintain higher protein synthesis and rapid growth of muscles. Not surprisingly, we noticed that a high percentage of amylose diets could promote the deposition of aspartate, alanine, leucine and proline in the longissimus dorsi muscle of goats. Similarly, a previous study has demonstrated that when goats are given high amylose diets, the plasma BCAA (leucine, isoleucine and valine) content tends to rise^(23)^. The concept has been established that it is feasible to increase the levels (including BCAA) of serum metabolites to regulate animal production in animal husbandry^(24–26)^. High leucine in plasma is usually thought to promote protein synthesis in skeletal muscle^(27,28)^. Our parallel studies have shown that diets with a high amylose ratio can improve goat growth performance. The increase of BCAA in the blood could promote muscle protein synthesis, so the goats fed high amylose diet should have stronger protein synthesis capacity and deposit more protein in muscle.

The AA that entered the small intestinal mucosa can be either used by the intestinal mucosa (AA are oxidised to provide energy, protein synthesis, etc.) or be transported into the blood by AA transporters to be used by other tissues. In this study, goats consumed a high proportion of amylose diets activated the mRNA expression of SLC38A1 and SLC38A2 in the rumen and ileum. Our data showed a high amylose diet up-regulated the protein abundances of AA transporters in the ileal mucosa, such as SLC3A2 and SLC38A9. SLC38A2 and SLC3A2 are involved in the transport of BCAA, which can pass extracellular glutamine and Na^+^ through the cell membrane into the cytoplasm^(29)^. Previous studies have clarified the importance of BCAA to promote protein synthesis by activating mTORC1^(30–32)^. The increased protein abundances of SLC38A1, SLC38A2 and SLC3A2 in the rumen and ileum of goats fed a high amylose ratio diet allowed more BCAA to be transported into the blood to maintain better growth performance.

The AA in the diets are the fuel source of the small intestinal mucosa, and as a substrate for protein synthesis in the small intestines, AA are essential for the growth and integrity of the small intestine mucosa^(33)^. The BCAA can be decomposed and utilised in the intestinal mucosa^(34)^. Our results indicate that an increase in the ratio of amylose in the diet could inhibit the mRNA expression of BCAA metabolising enzymes (ACADSB, BCAT1 and BCKDHB) in goat ileal mucosa. The first step in the catabolism of BCAA is the transfer of *α*-amino groups to *α*-ketoglutarate through the BCAT1 and BCAT2 enzymes^(35)^. Two crucial and irreversible reactions of BCAA catabolism require the participation of BCKDH complex and acyl-CoA dehydrogenase (ACAD)^(36–38)^. Furthermore, after feeding goats a high amylose diet, the metabolic enzyme activities of BCAA in the ileum mucosa also decreased, indicating that the high amylose diet could reduce the metabolism of amylopectin in the intestinal mucosa. This result supports our hypothesis: after goats consumed high amylose diets, the use of BCAA in the intestine would be reduced, increasing the content of BCAA entering the blood. The cause for this outcome might be that amylose had a slower digestion rate in the small intestines compared with amylopectin and could maintain a stable glycaemic index for a long time, so that the small intestine mucosa could preferentially use glucose for energy source, reducing part of the energy supply by oxidation of AA. Our results suggest that different starch types in the diet can affect the metabolism of AA in the intestinal mucosa of goats, especially for BCAA.

In mammals, activated mTOR plays a vital role in regulating protein synthesis through its downstream targets S6K1 and 4EBP1 in the body^(39,40)^. It has been reported that higher dietary amylose content reduces fat deposition and enhances protein deposition by altering the insulin/PI3K/Akt/mTOR signalling pathway in finishing pigs^(41)^. In the present study, the mRNA levels and protein abundances of genes related to protein synthesis (mTOR^(42)^, 4EBP1 and S6K1) were determined in goat muscle, and the mRNA and protein expression and phosphorylation ratio of mTOR, 4EBP1 and S6K1 were enhanced in the muscles of goats fed diets with a higher amylose:amylopectin ratio *v* goats fed a diet of low amylose:amylopectin ratio. This would be because high content of AA in plasma activated mTOR and enhanced the expression of its downstream targets S6K1 and 4EBP1. Similarly, a previous study has demonstrated that providing pigs with a higher percentage of amylopectin diet significantly reduced protein synthesis^(41,43)^. Our results further explained the possible mechanism by which high amylose diets to promote protein synthesis from the perspective of AA transport and metabolism (especially BCAA). The mTOR pathway may be the main factor regulating goat muscle protein deposition under different starch type diets.

A major finding was that higher dietary amylose contents not only elevated the mRNA levels and protein abundances of BCAA transporters (SLC38A1, SLC38A2) in the ileum of goats but also reduced the mRNA levels and activity of BCAA metabolising enzymes (ACADSB, BCAT, BCKDH), thus leading to an increase in the content of BCAA in the plasma. Furthermore, the high content of BCAA in the blood promotes protein synthesis in the longissimus dorsi muscle, which was likely mediated by the mTOR signalling pathway. The current results would help us better understand how dietary starch structure affects animal muscles growth by affecting AA metabolism.

## Supplementary material

For supplementary material accompanying this paper visit https://doi.org/10.1017/S0007114521002087. click here to view supplementary material

## References

[1] Chen MY , Ye JD , Yang W , et al. (2013) Growth, feed utilization and blood metabolic responses to different amylose-amylopectin ratio fed diets in tilapia (oreochromis niloticus). Asian-Australas J Anim Sci 26, 1160–1171. 25049897 10.5713/ajas.2013.13022 PMC4093233

[2] Zobel H (1988) Molecules to granules: a comprehensive starch review. Starch-Stärke 40, 44–50.

[3] Englyst HN & Hudson GJ (1996) The classification and measurement of dietary carbohydrates. Food Chem 57, 15–21.

[4] Englyst HN , Kingman S & Cummings J (1992) Classification and measurement of nutritionally important starch fractions. Eur J Clin Nutr 46, S33–S50. 1330528

[5] Gao X , Yu B , Yu J , et al. (2020) Effects of dietary starch structure on growth performance, serum glucose–insulin response, and intestinal health in weaned piglets. Animals 10, 543. 10.3390/ani10030543 PMC7142567 32213990

[6] Behall KM & Hallfrisch J (2002) Plasma glucose and insulin reduction after consumption of breads varying in amylose content. Eur J Clin Nutr 56, 913–920. 12209381 10.1038/sj.ejcn.1601411

[7] Rawles S & Lochmann R (2010) Effects of amylopectin/amylose starch ratio on growth, body composition and glycemic response of sunshine bass Morone chrysops × M. saxatilis. J World Aquacult Soc 34, 278–288.

[8] Roos S , Lagerlöf O , Wennergren M , et al. (2009) Regulation of amino acid transporters by glucose and growth factors in cultured primary human trophoblast cells is mediated by mTOR signaling. Am J Physiol Cell Physiol 297, C723–C731. 19587219 10.1152/ajpcell.00191.2009

[9] Broer S (2008) Amino acid transport across mammalian intestinal and renal epithelia. Physiol Rev 88, 249–286. 18195088 10.1152/physrev.00018.2006

[10] Drew MD , Schafer TC & Zijlstra RT (2012) Glycemic index of starch affects nitrogen retention in grower pigs. J Anim Sci 90, 1233. 21984722 10.2527/jas.2010-3458

[11] Van der Meulen J , Bakker J , Smits B , et al. (1997) Effect of source of starch on net portal flux of glucose, lactate, volatile fatty acids and amino acids in the pig. Br J Nutr 78, 533–544. 9389882 10.1079/bjn19970173

[12] Yang C , Chen D , Yu B , et al. (2015) Effect of dietary amylose/amylopectin ratio on growth performance, carcass traits, and meat quality in finishing pigs. Meat Sci 108, 55–60. 26047977 10.1016/j.meatsci.2015.05.026

[13] Doti S , Suárez-Belloch J , Latorre MA , et al. (2014) Effect of dietary starch source on growth performances, digestibility and quality traits of growing pigs. Livest Sci 164, 119–127.

[14] Li TJ , Huang RL , Wu GY , et al. (2007) Growth performance and nitrogen metabolism in weaned pigs fed diets containing different sources of starch. Livest Sci 109, 0–76.

[15] Li T-J , Dai Q-Z , Yin Y-L , et al. (2008) Dietary starch sources affect net portal appearance of amino acids and glucose in growing pigs. Animals 2, 723–729. 10.1017/S1751731108001614 22443597

[16] Giusi-Perier A , Fiszlewicz M & Rérat A (1989) Influence of diet composition on intestinal volatile fatty acid and nutrient absorption in unanesthetized pigs. J Anim Sci 67, 386–402. 2539347 10.2527/jas1989.672386x

[17] Yu M , Li Z , Rong T , et al. (2020) Different dietary starch sources alter the carcass traits, meat quality, and the profile of muscle amino acid and fatty acid in finishing pigs. J Anim Sci 11, 1–14. 10.1186/s40104-020-00484-9 PMC7412799 32782789

[18] Li F , Duan Y , Li Y , et al. (2015) Effects of dietary *n*-6: *n*-3 PUFA ratio on fatty acid composition, free amino acid profile and gene expression of transporters in finishing pigs. Br J Nutr 113, 739–748. 25704496 10.1017/S0007114514004346

[19] Huang J , Jiao J , Tan Z-L , et al. (2016) Inferring the skeletal muscle developmental changes of grazing and barn-fed goats from gene expression data. J Agric Food Chem 64, 6791–6800. 27561543 10.1021/acs.jafc.6b02708

[20] Livak KJ & Schmittgen TD (2001) Analysis of relative gene expression data using real-time quantitative PCR and the 2− ΔΔCT method. Methods 25, 402–408. 11846609 10.1006/meth.2001.1262

[21] Duan Y , Li F , Li L , et al. (2014) *n*-6: *n*-3 PUFA ratio is involved in regulating lipid metabolism and inflammation in pigs. Br J Nutr 111, 445–451. 23947577 10.1017/S0007114513002584

[22] Hundal HS & Taylor PM (2009) Amino acid transceptors: gate keepers of nutrient exchange and regulators of nutrient signaling. Am J Physiol Endocrinol Metab 296, E603–E613. 19158318 10.1152/ajpendo.91002.2008 PMC2670634

[23] Wang S , Wang W & Tan ZL (2016) Effects of dietary starch types on rumen fermentation and blood profile in goats. Czech J Anim Sci 61, 32–41.

[24] He J , Chen D , Yu B , et al. (2010) Metabolic and transcriptomic responses of weaned pigs induced by different dietary amylose and amylopectin ratio. PLoS One 5, e15110. 21152049 10.1371/journal.pone.0015110 PMC2994909

[25] Ren M , Zhang SH , Zeng XF , et al. (2015) Branched-chain amino acids are beneficial to maintain growth performance and intestinal immune-related function in weaned piglets fed protein restricted diet. Asian-Australas J Anim Sci 28, 1742. 26580442 10.5713/ajas.14.0131 PMC4647083

[26] Wiltafsky MK , Pfaffl MW & Roth FX (2010) The effects of branched–chain amino acid interactions on growth performance, blood metabolites, enzyme kinetics and transcriptomics in weaned pigs. Br J Nutr 103, 964–976. 20196890 10.1017/S0007114509992212

[27] Escobar J , Frank JW , Suryawan A , et al. (2005) Physiological rise in plasma leucine stimulates muscle protein synthesis in neonatal pigs by enhancing translation initiation factor activation. Am J Physiol Endocrinol Metab 288, E914–E921. 15644455 10.1152/ajpendo.00510.2004

[28] Escobar J , Frank JW , Suryawan A , et al. (2010) Leucine and *α*-ketoisocaproic acid, but not norleucine, stimulate skeletal muscle protein synthesis in neonatal pigs. J Nutr 140, 1418–1424. 20534881 10.3945/jn.110.123042 PMC2903301

[29] Nicklin P , Bergman P , Zhang B , et al. (2009) Bidirectional transport of amino acids regulates mTOR and autophagy. Cell 136, 521–534. 19203585 10.1016/j.cell.2008.11.044 PMC3733119

[30] Chen J , Su W , Kang B , et al. (2018) Supplementation with *α*-ketoglutarate to a low-protein diet enhances amino acid synthesis in tissues and improves protein metabolism in the skeletal muscle of growing pigs. Amino Acids 50, 1525–1537. 30167964 10.1007/s00726-018-2618-3

[31] Duan Y , Guo Q , Wen C , et al. (2016) Free amino acid profile and expression of genes implicated in protein metabolism in skeletal muscle of growing pigs fed low-protein diets supplemented with branched-chain amino acids. J Agric Food Chem 64, 9390–9400. 27960294 10.1021/acs.jafc.6b03966

[32] Escobar J , Frank JW , Suryawan A , et al. (2006) Regulation of cardiac and skeletal muscle protein synthesis by individual branched-chain amino acids in neonatal pigs. Am J Physiol Endocrinol Metab 290, E612–E621. 16278252 10.1152/ajpendo.00402.2005

[33] Wu G , Knabe D & Flynn N (2005) Amino acid metabolism in the small intestine: biochemical bases and nutritional significance. Biol Grow Anim 3, 107–126.

[34] Wu G (1998) Intestinal mucosal amino acid catabolism. J Nutr 128, 1249. 9687539 10.1093/jn/128.8.1249

[35] Tönjes M , Barbus S , Park YJ , et al. (2013) BCAT1 promotes cell proliferation through amino acid catabolism in gliomas carrying wild-type IDH1. Nat Med 19, 901–908. 23793099 10.1038/nm.3217 PMC4916649

[36] Alfardan J , Mohsen AW , Copeland S , et al. (2010) Characterization of new ACADSB gene sequence mutations and clinical implications in patients with 2-methylbutyrylglycinuria identified by newborn screening. Mol Genet Metab 100, 333–338. 20547083 10.1016/j.ymgme.2010.04.014 PMC2906669

[37] Wanders RJA , Duran M & Loupatty FJ (2012) Enzymology of the branched-chain amino acid oxidation disorders: the valine pathway. J Inherit Metab Dis 35, 5–12. 21104317 10.1007/s10545-010-9236-x PMC3249182

[38] Webb LA , Sadri H , Soosten DV , et al. (2019) Changes in tissue abundance and activity of enzymes related to branched-chain amino acid catabolism in dairy cows during early lactation. J Dairy Sci 102, 3556–3568. 30712942 10.3168/jds.2018-14463

[39] Gorissen SH & Phillips SM (2019) Branched-Chain Amino Acids (Leucine, Isoleucine, and Valine) and Skeletal Muscle. Nutr Skeletal Muscle 283–298.

[40] Panzhinskiy E , Culver B , Ren J , et al. (2019) Role of Mammalian Target of Rapamycin in Muscle Growth. Nutr Enhanced Sports Performance 251–261.

[41] Xie C , Li Y , Li J , et al. (2017) Dietary starch types affect liver nutrient metabolism of finishing pigs. Br J Nutr 118, 353–359. 10.1017/S0007114517002252 28901894

[42] Manjarín R , Columbus DA , Suryawan A , et al. (2016) Leucine supplementation of a chronically restricted protein and energy diet enhances mTOR pathway activation but not muscle protein synthesis in neonatal pigs. Amino Acids 48, 257–267. 26334346 10.1007/s00726-015-2078-y PMC4713246

[43] Deng J , Wu X , Bin S , et al. (2010) Dietary amylose and amylopectin ratio and resistant starch content affects plasma glucose, lactic acid, hormone levels and protein synthesis in splanchnic tissues. J Anim Physiol Anim Nutr 94, 220–226. 10.1111/j.1439-0396.2008.00902.x 19175452

