# Supplementary material for: Dietary amylose:amylopectin ratio influences the expression of amino acid transporters and enzyme activities for amino acid metabolism in the gastrointestinal tract of goats
Source: Br J Nutr. 2021 Jun 14;127(8):1121–31. doi: 10.1017/S0007114521002087 (PMC8980728; doi:10.1017/S0007114521002087)
Supplement: Supplementary file 1 [file S0007114521002087sup001.docx]

Table S1 Primers used in the present study

| Gene name | Primer | Sequence (5’-3’) | Size(bp) | References |
| --- | --- | --- | --- | --- |
| GRM7 | Forward | ACACTGCGTCAATCACAAACTG | 156 | ^(1)^ |
|  | Reverse | TTGCCGAATACTGGGAGGA |  |  |
| CASR | Forward | CTCTTCCCTGATTGCTATGCC | 119 |  |
|  | Reverse | TCCTGGGGTGGACTTTCTG |  |  |
| SLC1A1 | Forward | AGCGGAAAGTGACAGGCAGT | 155 |  |
|  | Reverse | TGGGCTTGCAATCCACTCCA |  |  |
| SLC1A3 | Forward | ATGGGACCGCCCTCTATGA | 105 |  |
|  | Reverse | CCGTGGCTGTGATGCTGAT |  |  |
| SLC1A4 | Forward | ATCTGGCTCGCAGACCCTTC | 150 |  |
|  | Reverse | GCATACGTCCGGAAAGCTGC |  |  |
| SLC7A5 | Forward | ACGGCTGCTGACGCCTGTAC | 123 |  |
|  | Reverse | GCCACGCAGAGCCAGTTGAAG |  |  |
| SLC7A10 | Forward | CGCCTTTGACTTCTGGATGA | 143 |  |
|  | Reverse | TGGCACGAGGTAGGTTCTTT |  |  |
| SLC38A1 | Forward | GTACGGCTGGCAGTCATTGT | 127 |  |
|  | Reverse | AGGTCACCGAGACATGACGA |  |  |
| SLC38A2 | Forward | AAAGCCATTATGCCGATGTAG | 166 |  |
|  | Reverse | GAAAGCCCAAGGATTCCACT |  |  |
| SLC7A1 | Forward | ATGAAGCAGATGAGCAGGGAC | 104 |  |
|  | Reverse | GCCTTCGTGGGCTTTGACT |  |  |
| SLC3A2 | Forward | CCTCGTCGTCGGCAACCTTG | 108 |  |
|  | Reverse | CCGAGAAGCAGCCGATGAACG |  |  |
| SLC38A9 | Forward | ATTTCCTTCACCACCAT | 230 | This study |
|  | Reverse | CTCCCACAATAAACAGAT |  |  |
| BCKDHA | Forward | CGTGGATGGCAATGATGTG | 184 | This study |
|  | Reverse | GTCCTGCTTGTCCCAGTAGTT |  |  |
| BCKDHB | Forward | ACCGCCATAGCAGAAAT | 198 | This study |
|  | Reverse | GCAGTGGGCAAAGAAAG |  |  |
| ACADS | Forward | CGCCTACATACCGTCTACCAG | 158 | This study |
|  | Reverse | TCGCCCATCTTCTTCACCT |  |  |
| ACADSB | Forward | ATTGCTGGGCTGTTCGTG | 157 | This study |
|  | Reverse | ATGTGATTGGGCAGGTGG |  |  |
| BCAT1 | Forward | TTTGCCCAATGTGAAGC | 147 | This study |
|  | Reverse | CGGAGGAGTTGCCAGTT |  |  |
| BCAT2 | Forward | CCGAGTGACAGAGCGTAAGA | 189 | This study |
|  | Reverse | TTGTGGAAGCGGAGGATAA |  |  |
| mTOR | Forward | CCTGTTCGTGGCTCTGAATGACC | 161 | ^(2)^ |
|  | Reverse | TTCTTCCAATGCCGCTGTGCTC |  |  |
| 4EBP1 | Forward | CTCACCTGTGACCAAGACGC | 101 | ^(2)^ |
|  | Reverse | GGTGATTCTGGCTGGCTTCC |  |  |
| S6K1 | Forward | CGCAGACGCCTGTGGAATACC | 95 | ^(2)^ |
|  | Reverse | TCGTATTGGAAGCGGTGCTGAAG |  |  |
| β-ACTIN | Forward | CTTCCAGCCTTCCTTCCTG | 111 | 101.4 |
|  | Reverse | ACCGTGTTGGCGTAAAGGT |  |  |

GRM7, glutamate receptor, metabotropic 7; CASR, calcium-sensing receptor; SLC1A1, solute carrier family 1 member 1; SLC1A3, solute carrier family 1 member 3; SLC1A4, solute carrier family 1 member 4; SLC7A5, solute carrier family 7 member 5; SLC7A10, solute carrier family 7 member 10; SLC38A1, solute carrier family 38 member 1; SLC38A2, solute carrier family 38 member 2; SLC7A1, solute carrier family 7 member 1; SLC3A2, solute carrier family 3 member 2; SLC38A9, solute carrier family 38 member 9; BCKDHA, branched-chain α-keto acid dehydrogenase E1, α polypeptide; BCKDHB, branched-chain keto acid dehydrogenase E1 subunit beta; ACADS, acyl-CoA dehydrogenase short-chain; ACADSB, acyl-CoA dehydrogenase short/branched-chain; BCAT1, branched-chain amino acid transaminase 1; BCAT2, branched-chain amino acid transaminase 2; mTOR, mammalian target of rapamycin; 4EBPI, 4E-binding protein 1; S6K1, ribosomal protein S6 kinases 1.

Table S2 Information of antibodies used for immunofluorescence

| Items | Source | Dilution |
| --- | --- | --- |
| Primary Antibody |  |  |
| Rabbit Anti-SLC38A9 antibody | Bioss, bs-19827R | 1:200 |
| Rabbit Ant-SLC3A2 Antibody | proteintech,15193-1-AP | 1:50 |
| Rabbit Anti-SLC38A1 Antibody | proteintech, 12039-1-AP | 1:50 |
| Rabbit Anti-SLC38A2 antibody | Bioss,bs-12125R | 1:50 |
| Secondary Antibody |  |  |
| CoraLite594 – conjugated Donkey Anti-Rabbit IgG(H+L) | proteintech,SA00013-8 | 1:200 |

Table S3 Information of antibodies used for western blot analysis

| Items | Source | Dilution |
| --- | --- | --- |
| Primary Antibody |  |  |
| Mouse Anti-4EBP1 antibody | Proteintech,60246-1-Ig | 1:1000 |
| Rabbit Anti-phospho-eIF4EBP1 | Bioss, bs-14550R | 1:500 |
| Rabbit Anti-S6K1 antibody | Proteintech,14485-1-AP | 1:2000 |
| Rabbit Anti-phospho-RPS6KB1 (Ser434) antibody | Bioss, bs-5671R | 1:500 |
| Rabbit Anti-MTOR antibody | Bioss, bs-1992R | 1:500 |
| Rabbit Anti-Phospho-mTOR (Tyr144) antibody | Bioss, bs-5329R | 1:500 |
| Rabbit Anti- GAPDH antibody | Proteintech, 10494-1-AP | 1:3000 |
| Secondary Antibody |  |  |
| HRP goat anti-mouse IgG | Proteintech, SA00001-1 | 1:5000 |
| HRP goat anti-rabbit IgG | Proteintech, SA00001-2 | 1:6000 |

Table S4 Effects of different dietary amylose/amylopectin ratios on AA profiles in rumen mucosal of goats

| Items | Treatments^1^ | | | SEM | *P*-value^2^ | |
| --- | --- | --- | --- | --- | --- | --- |
|  | T1 | T2 | T3 |  | L | Q |
| Asp | 1.17^b^ | 1.31^a^ | 1.19^ab^ | 0.027 | 0.68 | 0.03 |
| Thr | 0.58 | 0.64 | 0.57 | 0.014 | 0.85 | 0.05 |
| Ser | 0.58 | 0.63 | 0.58 | 0.013 | 0.78 | 0.08 |
| Glu | 2.06 | 2.26 | 2.05 | 0.048 | 0.87 | 0.05 |
| Gly | 1.47 | 1.54 | 1.45 | 0.037 | 0.83 | 0.34 |
| Ala | 1.12 | 1.18 | 1.07 | 0.026 | 0.45 | 0.11 |
| Val | 0.74 | 0.81 | 0.74 | 0.018 | 0.90 | 0.06 |
| Met | 0.29 | 0.31 | 0.27 | 0.009 | 0.25 | 0.09 |
| Ile | 0.58^b^ | 0.64^a^ | 0.57^b^ | 0.015 | 0.70 | 0.04 |
| Leu | 1.11^b^ | 1.25a | 1.13^ab^ | 0.028 | 0.71 | 0.02 |
| Tyr | 0.45 | 0.50 | 0.44 | 0.012 | 0.55 | 0.05 |
| Phe | 0.59^b^ | 0.66^a^ | 0.59^b^ | 0.015 | 0.87 | 0.04 |
| Lys | 1.06^b^ | 1.16^a^ | 1.04^b^ | 0.026 | 0.75 | 0.04 |
| His | 0.32 | 0.35 | 0.31 | 0.008 | 0.70 | 0.08 |
| Arg | 1.22^b^ | 1.36^a^ | 1.22^b^ | 0.031 | 0.97 | 0.04 |
| Pro | 1.01 | 1.11 | 1.03 | 0.025 | 0.68 | 0.09 |
| TAA^3^ | 13.86^b^ | 15.69^a^ | 14.25^ab^ | 0.340 | 0.63 | 0.02 |

a, b Means within a row with different superscripts differ (*P* < 0.05);

^1^Treatments were: T1 (normal corn 100%, high amylose corn 0%); T2 (normal corn 50%, high amylose corn 50%); T3 (normal corn 0%, high amylose corn 100%);

^2^L and Q represent linear and quadratic response to increasing amylose to amylopectin ratio;

^3^TAA, total AAs

**References**

1. D JWABC, D XZABC, C RWAB *et al.*(2020)*.* Replacing corn grain with corn gluten feed: Effects on the rumen microbial protein synthesis, functional bacterial groups and epithelial amino acid chemosensing in growing goats. *Anim. Feed Sci. Technol* **270**.

2. Xu L, Hanigan M, Lin X *et al.* (2019) Effects of jugular infusions of isoleucine, leucine, methionine, threonine, and other amino acids on insulin and glucagon concentrations, mammalian target of rapamycin (mTOR) signaling, and lactational performance in goats. *J DAIRY* SCI**102**, 9017-9027.
